# 肺癌多学科综合诊治现状及未来方向

**DOI:** 10.3779/j.issn.1009-3419.2024.102.15

**Published:** 2024-05-20

**Authors:** CHE Guowei, ZHOU Qinghua

**Affiliations:** ^1^610041 成都，四川大学华西医院肺癌中心/肺癌研究所; ^1^Lung Cancer Center/Lung Cancer Institute, West China Hospital, Sichuan University, Chengdu 610041, China; ^2^610041 成都，四川大学华西医院肺癌中心/胸外科; ^2^Department of Thoracic Surgery, West China Hospital, Sichuan University, Chengdu 610041, China

**Keywords:** 多学科诊疗, 临床决策, 诊治模式, 肺肿瘤, Multidisciplinary team, Decision making, Diagnosis and treatment mode, Lung neoplasms

## Abstract

尽管肺癌多学科诊疗（multidisciplinary team, MDT）模式能够提高患者的生活质量和生存预后，为何没有达到MDT诊疗模式所预期的目标呢?主要原因是肺癌MDT诊治模式相对滞后于各种治疗手段的进步。分析国内外肺癌MDT诊治最新研究成果，结合四川大学华西医院肺癌中心肺癌MDT诊治经验，本文将从以下几个方面对肺癌MDT的进展及未来发展方向进行讨论和总结：（1）肺癌MDT诊治模式的内涵和外延需要转变并适应新的诊治手段；（2）肺癌MDT诊治临床决策应当从“多学科会诊（multidisciplinary consultation, MDC）”向“MDT”转变；（3）肺癌MDT诊治过程要从“消防队模式”转向“防火墙模式”，并最终实行“个体化全程管理模式”；（4）肺癌MDT诊治路径要从“临时召集模式”向“肺癌单病种中心制模式”转变。

## Abstract (en)

Although multidisciplinary team (MDT) diagnosis and treatment model can improve the quality of life and survival prognosis of the patients, why does it not reach the expected goal of the MDT diagnosis and treatment model? The main reason is that the diagnosis and treatment mode of MDT in lung cancer lags behind the progress of various treatment methods. By analyzing the latest research results of MDT diagnosis and treatment of lung cancer at home and abroad, combined with the experience of MDT diagnosis and treatment of lung cancer in the Lung Cancer Center of West China Hospital of Sichuan University, this article will discuss and summarize the progress and future direction of MDT in lung cancer from the following aspects: (1) The connotation and extension of MDT diagnosis and treatment mode of lung cancer need to be changed and adapted to new methods of diagnosis and treatment; (2) The clinical decision making in the diagnosis and treatment of MDT in lung cancer should be transformed from "multidisciplinary consultation (MDC)" to "MDT"; (3) The diagnosis and treatment process of MDT in lung cancer should shift from "fire brigade mode" to "firewall mode", and finally implement "individualized whole-process management mode"; (4) The path of MDT diagnosis and treatment of lung cancer should be changed from "temporary convening mode" to "single disease center system mode of lung cancer".

肺癌发生发展机制和病理分子分型研究的进步、外科技术和内科治疗药物的进展，极大丰富和推动了肺癌的诊断和治疗，也使肺癌患者的生活质量和预后有所改善，但非小细胞肺癌患者5年生存率仍然只有20%左右，而小细胞肺癌更低^[1]^；这应该与肺癌细胞的异质性高、诊疗争议大、预后差异大密切相关^[2]^。研究^[3]^证明，肺癌多学科诊疗（multidisciplinary team, MDT）模式不但改善肺癌患者生活质量，也提高生存率，而目前的肺癌多学科诊治基本是多学科会诊（multidisciplinary consultation, MDC），会诊目的主要是针对肺癌患者临床诊治过程中特定问题需要其他专科的支持，讨论问题集中某一阶段的诊断和治疗方法，而对患者全程治疗、生活质量甚至预后关注不足；肺癌诊治模式创新需要从MDC向MDT转变，基于循证医学的多学科团队、综合最佳证据、专家经验和患者意愿，使患者诊治过程个性化、精准化；同时也可以优化医疗资源使用，提高诊疗的合理性，减少偏倚，最终通过临床诊疗决策的转变，使医患双方在多方面获益。肺癌MDT诊疗需要适应当前肺癌各种治疗手段的发展而做出相应的转变，强调结果导向（提高生存率和生活质量）、过程管理（准备、临床决策及时调整、治疗效果准确及时反馈、总结）、各学科（专科团队）相互支撑，从而使MDT诊治的目的和目标均达到预期效果。

## 1 肺癌MDT诊治的内涵和外延需要转变并适应新的诊治手段

肿瘤患者死亡的主要原因仍然是复发和转移，控制复发和转移均需要包括手术、内科药物（靶向药物、细胞毒药物和免疫药物等）、放疗和介入治疗（如血管栓塞或消融等）等在内的综合治疗，即MDT，尤其是肺癌，因其发病机制复杂、癌细胞异质性强等，单一学科或专家治疗难以达到理想的治疗效果^[4]^。国内肺癌多学科治疗无论从开始的年代和治疗效果均与国外同步，甚至在某些方面还优于国外^[5]^。这些成果的取得是一代又一代肺癌领域专家辛勤工作的结果，1993年周清华等^[6,7]^提出肺癌多学科综合治疗，提出将病理分期和分子分型用于指导肺癌的治疗，尤其是局部晚期肺癌外科手术在综合治疗中的作用及基础机制。吴一龙等^[8,9]^从基础到临床实践对肺癌综合治疗进行祥细论述。廖美琳等^[10⇓-12]^从2000年到2007年结合国内外肺癌治疗手段的变化，从不同侧面论述肺癌多学科治疗的重要性及临床意义。尤其是近几年医学技术和药物的进步，都极大地扩展了肺癌MDT的内容^[13⇓-15]^，《肺癌多学科团队诊疗中国专家共识》的发表，进一步确立并阐明了MDT诊疗在肺癌治疗中的作用及规范性，为我国肺癌的规范诊治起到了极大的推动作用^[5]^。

不同年代的肺癌多学科治疗都与当时的主要治疗手段相关，从以肺癌外科治疗为主的多学科治疗，到肺癌的多学科综合治疗。外科只是综合治疗的一部分，而不是为主，得益于肺癌病理分期、分子分型的内科药物治疗的进步^[7,11,14,15]^。不可否认的是，在这些阶段肺癌多学科治疗的内涵多数情况下仍是以“治病”为主，目标是明确诊断或/和治疗方案（基本上是临床有困难时才会进行MDT，更多是事后性）；而外延是几个相关专业（外科、肿瘤或呼吸）组成，讨论后转科或在原科室进行治疗，特征是结果难以反馈，治疗效果变异性大。若要充分发挥肺癌MDT的优势，内涵需要从“治病”向“治人”转变，目标是提高患者生存率和生活质量^[16]^；外延从几个相关专业向全医院所有相关专业延伸如诊断科室（如病理科、检验科等）、治疗科室[外科、内科（肿瘤或呼吸）等]、有助于改善生活质量相关科室（康复科、心理卫生中心、营养与膳食科、全程管理等），甚至需要相关研究部门如肺癌分子研究室或肿瘤研究所参与，对提高发病或治疗效果相关机制都有所帮助。总之，肺癌MDT治疗需要适应当下医学技术和药物等治疗手段的进步，即与时俱进。

## 2 肺癌MDT诊治临床决策要从“MDC”向“MDT”模式转变

国内外近40年的肿瘤MDT诊疗模式发展与临床实践均显示肿瘤MDT诊疗模式使肿瘤患者3年生存率从58%提高至66%^[17,18]^，缩短了约50%的治疗等待时间，减少了住院费用^[19]^，提升了患者治疗依从性和满意度。肺癌MDT在有些发达国家广泛开展且使每一位恶性肿瘤患者获得MDT诊疗的机会，讨论所有确诊肺癌病例的护理和诊疗^[20]^。尽管国内已形成各种肿瘤诊治的MDT专家共识，但MDT临床应用仍局限在肿瘤专科或综合性大医院，二级综合性医院多数未建立MDT^[21,22]^。部分医疗单位实施MDT诊疗不规范，事实上是以疑难病例讨论为主（即MDC），存在决策单一、执行率和随访率不高等不足。2019年，对全国已开展肺癌MDT诊疗模式的18家医院中的27位专家调查结果显示，国内肺癌MDT在指南遵循度、民主决策、会议记录、落地执行和反馈、患者随访等方面做得明显不够^[21]^。为促进肺癌MDT多学科合作，确保肺癌MDT诊疗模式的规范化、可持续性运行，真正地建立多学科团队的合作机制，2020版《肺癌多学科团队诊疗中国专家共识》一方面在建立中国肺癌MDT的标准和运行方面提供了理论和方法支持，同时也为肺癌患者提供了最优化的MDT诊疗方案，并立足于提高肺癌患者的生存预后及生活质量^[23]^；另一方面也有利于肺癌临床研究所需要的入组患者并提升研究质量，有助于完善中国肺癌MDT标准并普及推广。

现在临床普遍开展的肺癌MDT，主要还是在肺癌患者诊疗过程中出现疑问、困难、甚至是需要相应专科配合治疗时发起的，特征是被动和一定指向性，事实是一种MDC模式，应该是MDT初级阶段。肺癌MDC向MDT诊疗模式转变需要有固定团队和相应诊疗机制的建立，肺癌MDT诊疗模式有利于促进临床跨学科交流、融合和支撑，利于临床研究的开展，有助于青年人才的成长和培养，并促进医疗资源的优化配置。MDT诊疗模式在临床上的应用和实施，可提高疑难问题的决策效率，并调动工作人员积极性和增进心理健康^[24]^。

肺癌MDT诊治有别于MDC的临床决策首先是基于患者获益（生存率和生活质量）的最佳综合治疗方案；其次准确的治疗前诊断和分期；再次是选择的治疗方案需要综合既有的证据（符合国内外肺癌诊疗指南、指导原则）并根据患者个体情况进行调整（尤其是患者和/或家属意愿甚至治疗倾向性、积极性、经济条件与医保情况等）；最后需要使用非标准治疗方案时，必须理由充分且如实记录，患者和家属治疗意愿可能与专家意见相左，但在决策执行时必须充分尊重^[25]^。若做到以上几点，肺癌的临床诊治工作中就会逐渐从MDC转变为MDT。

## 3 肺癌MDT诊治过程要从“消防队模式”转向“防火墙模式”，并最终实现“个体化全程管理模式”

“消防队模式”主要特点就是事后性，当问题发生时召集相关人员紧急处理，更加强调解决当前和急症问题，这也可能是目前肺癌MDC的主要现状，也符合目前的国情和工作现实。但随着国家经济发展、医学理论和技术的进步，医学模式由“生物医学模式”（从物理学角度考虑人的健康和疾病的发生、发展和转归）转变为“生物心理社会（bio-psycho-social medical model）模式”（以人类的疾病谱以及健康观念的变化为依据）^[26]^。生物心理社会医学模式更加关注“自然的人”（人的状态和人所处的环境），生物心理社会模式实现了对生物医学模式（只关注疾病）的超越。医学模式的转变，也需要临床决策随之进行变化，从被动治疗模式（消防队模式）向主动预防治疗模式（防火墙模式）转变，并最终达到“个体化全程管理模式”（预防、治疗和管理并重）^[27]^。

“个体化全程管理模式”主要特点：一是基于“分子分期”和“分子分型”的“个体化”MDT治疗；二是从时间轴上看是全生命周期健康管理，需要各个专业（医、护、康及心理）全面照护，同时关注人及人的状态和环境；三是疾病和健康同时管理，更加重视预防，治疗时也要预防可能导致的医疗损害。肺癌MDT诊疗应该是从全程介入，对每一位需要MDT的患者制定详细的“个体化”诊治方案，并对可能出现的问题有处理对策，治疗过程中的信息及时反馈，团队成员应随时关注并提醒、预防或处理相关问题，随时再次发起MDT讨论。其次肺癌治疗过程中，也要关注治疗过程中可能导致的医疗损害或不良事件，对于较严重的不良事件也需要预防，如肺穿刺或脓肿型肺癌手术过程中出现气胸或大出血，外科医生应及时处理，当然这需要有预案和团队支撑。问题、讨论、收集、反馈、及时处理，这对MDT团队及场所要求较高，但临床效果是最好的。“个体化全程管理模式”对人才培养与临床研究以及提高医患满意度有极大帮助；MDT医护团队成员治疗患者过程中的心理压力会显著减轻，而患者对治疗方案心理上的接受度及效果都会显著提高，同时年轻医生准备资料、管理患者和处理紧急事件的能力都会得到极大提升。

“消防队模式”向“个体化全程管理模式”的转变也可以从根本上消除肺癌患者治疗前从各种渠道得到的大量不准确、零碎和不严谨的信息所造成种种心理焦虑。患者这种先入为主的焦虑状态可以通过MDT团队决策得到解决。因此，肺癌“个体化”全程管理MDT模式的意义在于实时质控、动态管理，而不是仅仅事后质控；既治病，也治人（改善人的生存状态，更重要的是防病和促进健康）。

## 4 肺癌MDT诊治路径要从“临时召集模式”向“肺癌单病种中心制模式”转变

国外发达国家的肺癌MDT模式不一定适合中国，国内模式主要是以MDC的虚拟中心制模式为主，多数开展肺癌MDT的医院都有固定的团队、固定的时间和场所、会诊信息的记录及治疗过程的反馈等，某些医院还有相应的激励政策，这些机制符合国情也有利于肺癌MDT的开展，但这些都属于“临时召集模式”或“虚拟中心制模式”。“临时召集模式”或“虚拟中心制模式”符合国情，便于开展，但也存在过程繁琐、团队成员变动大、患者等待治疗时间长，甚至绩效或价值体现不充分、医疗过程及时反馈难等不足，尤其是门诊MDT需要多学科时，出现专家排班及变动大的难题。若要克服肺癌MDT运行过程中的这些难题，实体“肺癌中心制模式”是发展的方向。

虚拟或实体“中心制模式”都需要医院整体决策（如学科融合、人员与绩效统一管理等）。肺癌MDT诊疗运行过程中，“实体肺癌中心制模式”更有助于MDT的开展与运行，四川大学华西医院于2014年成立四川大学华西医院肺癌中心（由胸外科、呼吸与危重医学科、肿瘤化疗与放疗科、康复科、影像诊断团队、护理团队及肺癌分子研究所组成），人员、交排班、绩效统一管理，真正做到学科融合、相互支撑、共同发展。肺癌中心的患者都能适时进行“个体化”MDT方案的制定，也使MDT优势充分发挥。经过近10年的发展，已形成了完整的肺癌MDT诊治特色和体系。

四川大学华西医院肺癌中心肺癌MDT诊治目标是创新模式、精准治疗；特色是全员（医生、护士、研究生、规培生、进修生）参与、结果导向、过程管理、学科支撑和相互融合。肺癌MDT体系体现在医院间、医院内部和科室内部层面，线上及线下MDT。医院间：四川大学华西医院医联体或重症肺癌联盟医院线上MDT（随时申请和每月固定一次相结合）和线下团队间MDT每月一次，肺癌康复诊疗MDT（社区医院及康复中心等）。医院内部：科室人员积极融入医院整体MDT规划，如参与肺结节MDT（呼吸与危重医学科为主导）、肺癌全程管理MDT（全程管理中心）。科室内部：肺癌MDT整体推进，肺癌康复MDT（肺癌中心、康复科、麻醉科、营养科和重症监护病房）；肺癌门诊MDT（每周三次）、病房MDT（每日一次）、互联网线上MDT（随时开展）。同时为总结并分享肺癌MDT的经验，科室定期召开中国肺癌多学科治疗论坛和华西肺癌MDT论坛。华西肺癌MDT体系的建立，医、护、患“不放弃”治疗成为坚定的信念，团队相互支撑，疾病的每个关键阶段都果断采取必要的措施，是许多患者治疗成功的关键。四川大学华西医院肺癌中心MDT团队遵循“以患者为中心”的理念，以“团队融合，相互支撑，精准施治，敢于担当”为宗旨，以改善患者的生活质量和提高生存率为目标。

肺癌MDT的合理运行不但可以制约片面的个人诊疗决策，合理并优化医疗资源的利用，提高不同角色医护人员在诊疗决策中的参与度，也能提高患者从肺癌MDT诊疗中的临床获益^[28]^ ；也有助于提升团队医疗水平，讨论临床问题，加快临床试验入组效率，降低医疗纠纷，最终使医患双方均受益^[29]^。中国肺癌多学科诊治在一代又一代专家们的努力下已取得了很大的发展，共识及各个中心经验的积累必将推动肺癌MDT的规范和普及，也必将造福于每位肺癌患者。
